# Chemokine-Releasing Microparticles Improve Bacterial Clearance and Survival of Anthrax Spore-Challenged Mice

**DOI:** 10.1371/journal.pone.0163163

**Published:** 2016-09-15

**Authors:** Taissia G. Popova, Allison Teunis, Virginia Espina, Lance A. Liotta, Serguei G. Popov

**Affiliations:** 1 Center for Applied Proteomics and Molecular Medicine, Department of Molecular Microbiology, School of Systems Biology, George Mason University, Manassas, VA, 20110, United States of America; 2 National Center for Biodefense and Infectious Diseases, Department of Molecular Microbiology, School of Systems Biology, George Mason University, Manassas, VA, 20110, United States of America; CCAC, UNITED STATES

## Abstract

In this study the hydrogel microparticles (MPs) were used to enhance migration of neutrophils in order to improve outcome of anthrax infection in a mouse model. Two MP formulations were tested. In the first one the polyacrylamide gel MPs were chemically coupled with Cibacron Blue (CB) affinity bait. In the second one the bait molecules within the MPs were additionally loaded with neutrophil-attracting chemokines (CKs), human CXCL8 and mouse CCL3. A non-covalent interaction of the bait with the CKs provided their gradual release after administration of the MPs to the host. Mice were challenged into footpads with *Bacillus anthracis* Sterne spores and given a dose of MPs a few hours before and/or after the spores. Pre-treatment with a single dose of CK-releasing MPs without any additional intervention was able to induce influx of neutrophils to the site of spore inoculation and regional lymph nodes correlating with reduced bacterial burden and decreased inflammatory response in footpads. On average, in two independent experiments, up to 53% of mice survived over 13 days. All control spore-challenged but MP-untreated mice died. The CB-coupled particles were also found to improve survival likely due to the capacity to stimulate release of endogenous CKs, but were less potent at decreasing the inflammatory host response than the CK-releasing MPs. The CK post-treatment did not improve survival compared to the untreated mice which died within 4 to 6 days with a strong inflammation of footpads, indicating quick dissemination of spores though the lymphatics after challenge. This is the first report on the enhanced innate host resistance to anthrax in response to CKs delivered and/or endogenously induced by the MPs.

## Introduction

Spatial and temporal concentration gradients of chemoattractants direct many biological processes involving leukocyte migration during development, regulation of homeostasis and ongoing immune responses within lymphoid organs and peripheral tissues. Chemokines (CKs) are an important class of these chemoattractant molecules [1]. Immune cells expressing the appropriate CK receptors typically migrate up chemotactic gradients of CKs toward their source to participate in the immune responses, such as presentation of antigens or elimination of pathogens and tumors. Manipulation with chemotaxis for therapeutic purposes opens new possibilities to design more effective vaccine adjuvants, anti-tumor reagents, anti-inflammatory and anti-microbial treatments.

Previous studies demonstrated application of the controlled-release nanomaterials loaded with CKs for the recruitment of immune cells applicable to basic studies and therapeutic applications [2–5]. Also, there are reports that non-functionalized nanoparticles and microparticles (MPs) of several kinds are themselves capable of eliciting the immune responses such as production of cytokines and activation of neutrophils raising questions of their potential utility for a stimulation of host defenses as well as their safety upon a prolonged contact with normal tissues [6]. It was recently proposed using a new class of CK-releasing MPs consisting of a non-toxic polyacrylamide hydrogel covalently coupled with a variety of affinity baits such as dyes of different chemical nature [7]. The MPs can be loaded with substances of interest for a reversible release from the baits at a controlled off-rate and dose depending on the property of the bait-ligand pair [8]. The hydrogel structure protects the loaded cargo to assure preservation of its function from degradation in the complex biological environment.

We recently applied the bait-hydrogel MP technology to increase an influx of neutrophils into draining lymph nodes (LNs) of mice [8]. For this purpose the MPs containing the Reactive Blue-4 bait were loaded with the neutrophil-attracting CKs, a mixture of human IL-8/CXCL8 and murine MIP-1α/CCL3 [9,10]. Inoculation of these CK-loaded MPs into footpads of mice enhanced the number of neutrophils migrating to the sites of injection and the regional popliteal LNs. Based on these results, in the current study we chose to test the MP-based CK gradient remodeling approach in the course of infectious disease.

The CK-related pathologies have been documented in the course of many infections; however, the field of MP-based CK delivery in infectious disease remains unexplored. We hypothesized that enhancing the recruitment of immune cells in the course of infection would provide new opportunities for therapeutic interventions such as boosting the innate response in the lymphatics resulting in the increased bactericidal effect beneficial to the host. We chose experimental anthrax caused by the administration of *B*. *anthracis* (*B*.*a*.) spores into footpads of susceptible mice as a model system to manipulate for the first time the process of immune trafficking using MPs and evaluate its contribution to the outcome of disease. During all forms of anthrax infection (inhalational, cutaneous, and gastrointestinal) the *B*.*a*. first spreads *via* lymphatics before appearing in the bloodstream independently of the spore entry route. Tissue macrophages and DCs uptake the spores from the site of exposure and deliver them within a few hours to the draining LNs, where anthrax lethal and edema toxins (LT and ET) expressed by germinating spores disrupt functions of DCs, including the ability of DCs to release inflammatory cytokines and attract monocytes and neutrophils [11]. Bacteria then quickly multiply in the LNs causing hemorrhagic lymphadenitis, gain access to the circulation and disseminate [11,12]. In the case of footpad challenge the migrating spores are known to follow a single route and first accumulate in the popliteal LN. This feature of the murine lymphatics makes a popliteal LN a convenient target for the MP-based intervention. We previously showed that the footpad-injected MPs are quickly delivered to the popliteal LNs where they can exert their effect on trafficking and activation of host phagocytic cells known to play key roles in the propagation of anthrax in the host.

Treatment of anthrax represents a significant clinical challenge. Half of patients who develop systemic disease are expected to die, despite improvements in modern therapeutic measures. The establishment of *B*.*a*. infection in the lymphatic system constitutes a critical point at which pharmacological intervention could prevent transition to a lethal systemic stage of disease. However, many aspects of the immune trafficking in anthrax remain insufficiently understood, and current antibiotics poorly penetrate the LNs. Viable bacilli can remain in patient's LNs until death while the bloodstream is sterile after antibiotic therapy [13]. Therefore, novel treatments that effectively target the complex molecular processes caused by bacteria in the lymphatics are needed. Toward this goal we decided to explore the effect of enhanced neutrophil trafficking on the outcome of anthrax in mouse model. Neutrophils can be readily mobilized in response to CK stimulation and are capable of efficient innate protection in several experimental models of bacterial and fungal infections. However, in anthrax the role of neutrophils in either promoting or suppressing host immunity is controversial. We previously showed that MPs induce rapid neutrophil recruitment to the primary site of inoculation and regional popliteal LNs [8]. This response can be enhanced by the pre-loading of MPs with CKs. In this study, we used the MPs coupled with Cibacron Blue (CB) dye with the CK binding properties characterized in our previous experiments. Mice were administered with CB-coupled MPs (further referred as MPs) loaded with CXCL8 and CCL3 before and/or after the footpad subcutaneous challenge with *B*.*a*. spores. Pre-treatment of mice with the CK-loaded MPs increased neutrophil migration to draining LNs in infected mice, reduced the bacterial burden and the inflammatory response in footpads, restricted the systemic spread of the bacilli, and ultimately promoted survival. The MPs themselves without loaded CKs contributed to the protective effect of pre-treatment. However, the MP treatment shortly after spore challenge did not increase survival, in line with our previous observations on the quick systemic spread of the spores.

## Materials and Methods

### Materials

Cibacron Blue F3G-A (CB) was purchased from Polysciences, Inc. (Warrington, PA, USA). The carrier-free recombinant CKs from BioLegend (San Diego, CA, USA) were a mouse CCL3 (MIP-1α), and a human CXCL8 (IL-8). Cell culture media and reagents were purchased from Mediatech, Inc. (Manassas, VA, USA). The CyQUANT^®^ NF Cell Proliferation Assay Kit was from ThermoFisher Scientific (Waltham, MA, USA). Endotoxin-free water was from Life Technologies (Fredrick, MD, USA). *B*. *anthracis* Sterne strain 34F2 was from Colorado Serum Co. α-*B*. *anthracis* serum recognizing the vegetative bacterium was raised in rabbits after a subcutaneous spore challenge. It was shown by us to recognize a vegetative form of the bacterium.

### Synthesis of the crosslinked MPs with chemically-coupled dye affinity bait and quantification of endotoxin content

Poly(N-isopropylacrylamide) MPs containing co-polymerized allylamine were prepared *via* precipitation polymerization as described [8]. N-isopropylacrylamide (9.0 g) and N-N′-methylenebisacrylamide (0.28 g) were dissolved in 250 ml of water, and the solution was partially degassed by vacuum filtration through a 0.45 μm nylon filter. The filtered solution was purged with nitrogen at room temperature upon stirring for 15 min. Allylamine (670 μl, 12 μmoles) was then added and the solution was purged with nitrogen for another 15 min and then heated to 75°C. After stably reaching 75°C, polymerization was initiated with the addition of potassium persulfate (0.1 g) in 1 ml of water. The reaction was maintained with stirring under nitrogen for 3 h. After this time, the reaction was allowed to cool to room temperature overnight with stirring under nitrogen. The MPs were then pelleted by centrifugation for 20 min at 23°C at 16,000 g and re-suspended in 300 ml of water. This centrifugation-dispersion process was repeated for a total of 5 times. The N4 Plus PCS Submicron Particle Analyzer (Beckman Coulter) was used to determine the particle size (500–600 nm) and the polydispersity index in water, which was in the interval of 0.2 to 0.5.

Cibacron Blue F3G-A (CB), the reactive triazine dye, was immobilized via direct reaction with the amine group of the MP allylamine units, displacing the chlorine on the di-substituted triazine ring of the dye. The coupling of the dye was performed under sterile, endotoxin-free conditions. The MPs were re-suspended in the endotoxin-free water and incubated with the dye (2 g CB dye per 325 ml of total solution) for 36 h at room temperature. After dye incorporation, the MPs were washed six times using tissue culture grade PBS diluted 1:3 with endotoxin-free water. The absence of bacterial contaminants was demonstrated by plating 100 μl of final MP suspension onto Luria Broth agar plates and incubating them at 37°C for 48 h. A few drops of chloroform as a bactericidal agent were added to the final batch of MPs stored at 4°C.

The endotoxin content of MPs was measured with the Pierce *Limulus* Amoebocyte Lysate (LAL) Chromogenic Quantitation Kit (ThermoFisher) according to the manufacturer’s protocols. The *E*. *coli* endotoxin standard provided in the kit was serially diluted with the endotoxin-free water. The supernatant from MP suspensions prepared under the endotoxin-free was collected for analysis. Supernatant and diluted standards were incubated with the kit’s synthetic substrates at 37°C for 6 min, and the endotoxin-dependent proteolysis of the substrate was measured at 405 nm as the amount of released p-nitroaniline after quenching of reaction with acetic acid. MPs prepared using the endotoxin-free conditions contained 0.14 EU/ml of endotoxin.

### Loading of MPs with CKs

Loading of the MPs with CKs for *in vitro* assays was accomplished by incubating CB MPs (10% wet *v/v*) with 1 μg/ml of indicated CK in 1/3 PBS at 4°C overnight. The buffer was supplemented with 100 U/ml of penicillin and 100 μg/ml streptomycin (pen/strep) to prevent bacterial contamination. The MPs were pelleted, supernatants removed, and the MP pellets were re-suspended in the culture medium for chemotactic assays as described below. The suspensions of CK-loaded NPs used for animal injections were prepared by incubating CB MPs (10% wet *v/v*) in PBS with a mixture of CXCL-8 and CCL3 (1 μg/ml each) at 4°C overnight. The suspensions were brought up to room temperature and injected into footpads of mice as described for animal challenge experiments below.

### In vitro chemotactic assays for immune cell migration analysis

*In vitro* chemotactic assays were conducted in the 96-well format. The transwell inserts incorporating tissue culture-treated polycarbonate membrane filters (8.0 μm pores) from Neuro Probe, Inc. were used. Cell migration of monocytic human THP-1 cells from ATCC, Manassas, VA, was measured. The bottom chambers of the transwell plates contained 300 μl of complete serum-free medium (CSFM) from Mediatech (Manassas, VA) with or without CCL3. The CK solutions were assayed at several concentrations up to 100 ng/ml. The MP-bound CCL3 was assayed after its release from the MPs. For this purpose, the MPs loaded with CCL3 as described above were resuspended in a 10-fold greater volume of CSFM at 37°C for 3 h. The MPs were removed by centrifugation and the supernatants containing released CK were transferred into bottom wells of the transwell plate. Dilutions of supernatants were prepared in the transwell plate immediately prior to assay performance. To increase the chemotactic responsiveness of migrating cells, the latter were starved in CSFM at 37°C, 5% CO_2_ for 1 h prior to assay. The serum-starved cells (50 μl of 1.28x10^7^ cells/ml) were added to the top chambers. Cell migration after 4 h at 37°C, 5% CO_2_ was enumerated by the DNA-binding fluorescent dye using the CyQUANT NF Cell Proliferation Assay Kit (Thermo Fisher Scientific, USA) according to the manufacturer’s protocols. Briefly, cells from the bottom chambers were pelleted by centrifugation, incubation medium was removed, and the cells were permeabilized using the kit’s dye delivery reagent to allow the dye to associate with nuclear DNA. After 10-min incubation at room temperature in the dark, the stable fluorescence of DNA-dye association was measured using a fluorimeter at 485/538 nm. Fluorescence intensity was converted to the fraction of migrated cells using the calibration curves obtained with the known number of cells.

The number of cells trapped on the membrane that were unable to fully migrate to the bottom chamber was estimated after staining with Crystal Violet. For staining, the membranes were rinsed three times with PBS and fixed with methanol for 15 min, incubated with 3% Crystal Violet stain (Becton Dickinson, MD, USA) at room temperature for 15 min, washed three times with water, and air-dried. The cells were counted under microscope. Less than 0.1% of the total cell numbers were retained on the membranes, and were therefore not considered for calculations.

### Animal challenge experiments

All animal experiments were conducted under protocol #284 approved by George Mason University’s Institutional Animal Care and Use Committee. Groups of female 6-8-week-old DBA/2 mice (Jackson Labs) were challenged with toxinogenic, non-encapsulated vaccine strain *B*. *anthracis* Sterne 34F2 spores in PBS into each hind footpad as previously described [8]. At certain times before and/or after the spore challenge, mice received 50 μl intradermal footpad injections of MPs with or without loaded CKs, as well as the CKs only, by the same route as spores. The number of mice per group (from 5 to 10) and the spore doses (from 0.4x10^6^ to 4x10^6^ in 20 or 50 μl of PBS) are indicated in the corresponding figure legends. The animals were monitored once or twice daily and were euthanized by carbon dioxide asphyxiation if one or more of the following criteria were met: (1) Rough hair coat, hunched posture, distended abdomen, or lethargy if debilitating, (2) Respiratory distress (dyspnea) or cyanosis, (3) Central nervous system signs such as head tilt, tremors, spasticity, seizures, circling, or paresis, (4) Persistent lateral recumbency, (5) Impaired mobility interfering with eating, drinking, or ambulation. Analgesics were not administered due their potential interference with the infectious process. All injections were performed under anesthesia using isoflurane inhalation for chemical restraint and reduction of stress. Thirty minutes before euthanasia, the animals were injected with 20 μl of 1% Evans Blue dye in PBS into both hind footpads for visualization of LNs. One popliteal LN and one footpad from each animal were placed in 10% neutral buffered formalin solution for immunohistological analysis. The formalin-incubated tissues were embedded in paraffin blocks, sliced into 5 μm sections, and mounted onto glass slides for staining. For enumeration of bacterial titers, the spleen and another popliteal LN from each infected animal were homogenized using frosted glass slides and suspended in PBS. Volumes of 10 μl and 100 μl of homogenized tissue suspensions were plated onto Luria Broth agar plates and incubated at 37°C overnight. Semi-quantitative scores of footpad inflammation and edema were assigned immediately prior to Evans Blue dye injection as: 0 = no visible signs, 1 = initial signs of swelling and light redness in the footpad, 2 = prominent swelling and redness partially extending from the footpad to the ankle, 3 = strong swelling and redness extending to the whole ankle, 4 = extensive swelling and redness beyond the ankle. The statistical significance of inflammation scores was determined using a two-tailed Mann-Whitney U test available online at http://www.socscistatistics.com/tests/mannwhitney/. Mortality curves were compared using the Log Rank Test Statistic, which was calculated using MedCalc statistical software.

### Immunohistochemical analysis

Slides used in immune cell staining were subjected to antigen retrieval in sodium citrate buffer (pH 6) for 40 min at 95°C followed by incubation for 20 min at room temperature. For slides stained with the anti-bacterial serum, the incubations at 95°C and room temperature were 20 min each. All slides were stained with antibodies using a Dako autostainer and counter-stained with Mayer’s hematoxylin. To detect the presence of neutrophils, tissue sections after antigen retrieval were incubated with the primary biotin-labeled anti-Ly-6G antibody (Biolegend, USA) followed by the Dako CSA streptavidin-biotin-peroxidase complex. Antibody staining was completed with a 5-minute incubation with 3,3’-diaminobenzidine tetrahydrochloride and followed with counter-staining using Mayer’s hematoxylin. For staining using the non-biotinylated antibodies or immune serum against *B*. *anthracis*, the secondary reagent was the anti-rabbit EnVision+ HRP-Labeled Polymer (Dako, USA).

## Results

### CKs released from MPs retain their chemotactic activity in the *in vitro* cell migration assays

**Fig 1:**
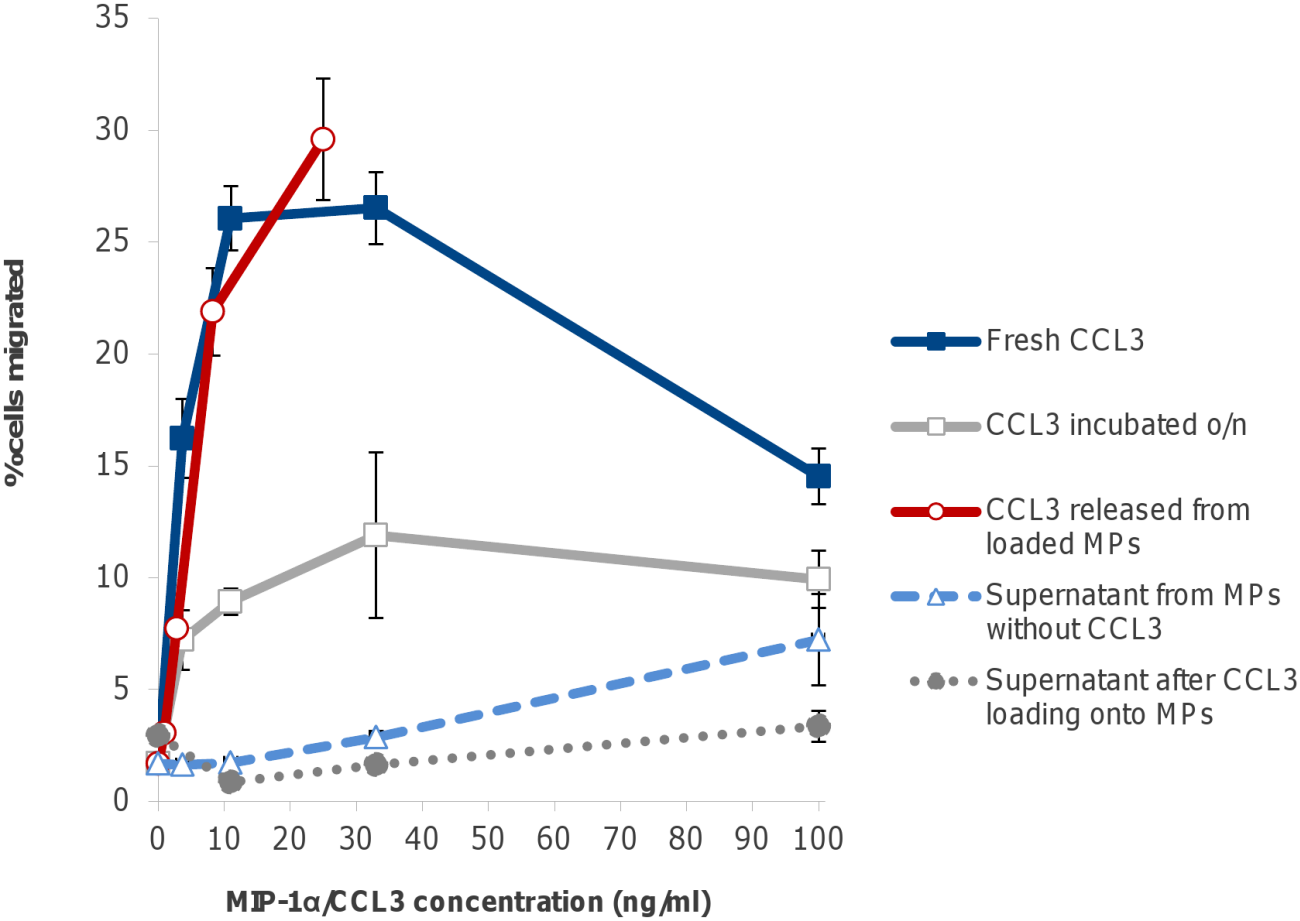
Chemotactic activity of CCL3 after release from MPs with THP-1 cells in a transwell format. The MP-bound CCL3 was assayed after its release from the MPs in CSFM at 37°C for 3 h. The MPs were removed by centrifugation and the supernatants containing released CK were transferred into bottom wells of the transwell plate. The serum-starved cells in CSFM were added to the top chambers. Cell migration to the bottom chamber was enumerated after incubation for 4 h at 37°C, 5% CO_2_. Values on the x-axis correspond to the concentrations calculated from the total amount of CCL3 used to prepare solutions (solid lines with open and closed squares) or the estimated amount of CCL3 released from the MPs during 3-h incubation (solid line with open circles). Dashed line represents the chemotactic activity of supernatants after the control incubations of MPs without CCL3 in the amounts used for the standard curve. Dotted line corresponds to the chemotactic activity left in solution after MPs were loaded with the indicated concentrations of CCL3.

We previously showed that the MPs with coupled CB dye readily bind CKs and release them with a half-life of several hours after dilution with a fresh buffer [8]. However, the biological activity of the released CKs was not demonstrated. Therefore, we tested the chemotactic activity of the MPs loaded with selected CKs in comparison with the freshly-prepared solutions of soluble CKs. MPs were co-incubated with CKs at 4°C overnight and the loaded MPs pelleted for analysis by centrifugation to separate them from the supernatants. To demonstrate the chemotactic activity of the CKs after their release from the loaded MPs, the latter were re-suspended in CSFM and incubated for 3 h at 37°C. The MPs were pelleted and the supernatants tested in the Boyden-type transwell assay. Fig 1 shows representative results obtained with CCL3-loaded MPs and THP-1 test cells which were highly responsive to CCL3. Similar experiments were carried out with CXCL8 and the U937 cells (the THP-1 cells did not respond to CXCL8; not shown). The freshly-diluted CCL3 displayed a typical dose-response standard curve with a maximal activity in the range of 10 to 30 ng/ml. As expected, CCL3 loaded onto MPs and partially released before the assay showed only a fraction of the chemotactic activity anticipated in the case of a full CCL3 release. According to the off-rate determined in our previous study (t_1/2_ 7.4 h) [8], the 3-h incubation estimated to dissociate about 25% of the bound CK. When plotted against the estimated amount released, the chemotactic activity of CCL3 in Fig 1 overlapped with the standard curve, indicating no substantial loss of the CK activity due to the MP binding and release. In comparison, CCL3 incubated without MPs in the conditions of CK loading lost a substantial part of its activity, likely due to its aggregation in a diluted solution [14].

### MPs influence the inflammatory response at the site of *B*.*a*. infection and survival of the spore-challenged mice depending on the CK load and the time of administration

Injection of *B*.*a*. spores into mice footpads results in bacterial proliferation at this site [15]. The MPs injected by the same route follow the migration of spores [16] and therefore can be used to manipulate with the local response to infection through the delivery of MPs’ cargo. The released CKs are then expected to form chemotactic gradients attracting the corresponding immune cells. It’s also likely that MPs themselves can play an immune-modulating role through the induction of endogenous CK/cytokine release by the host cells [6].

To assess the effect of MPs and loaded CKs on the outcome of *B*.*a*. infection, six groups of animals challenged with equal numbers of spores into each of the hind footpads were treated as described below with equal doses of CKs per injection. Spore-challenged animals in Group 1 served as untreated controls. Group 2 received MPs only, first at 24 h before (pre-treatment) and then at 4 h and 24 h after infection (post-treatment). Group 3 was pre-and post-treated with a mixture of the soluble neutrophil-attracting CKs, CXCL8 and CCL3 without MPs. These groups allowed for evaluation of the individual influences of MPs without loaded CKs, and soluble CKs without MPs on the outcome of infection.

Groups 4–6 compared the effects of pre-treatment, post-treatment and their combination. Group 4 was only pre-treated with the CK-loaded MPs, while Group 5 was pre- and post-treated with the same MPs. Finally, Group 6 received CK-loaded MPs as a post-treatment only. The survival was monitored for 13 days. Mice were assigned semi-quantitative scores for the inflammation and swelling seen in the challenged footpads during the course of disease as described in Materials and Methods.

**Fig 2:**
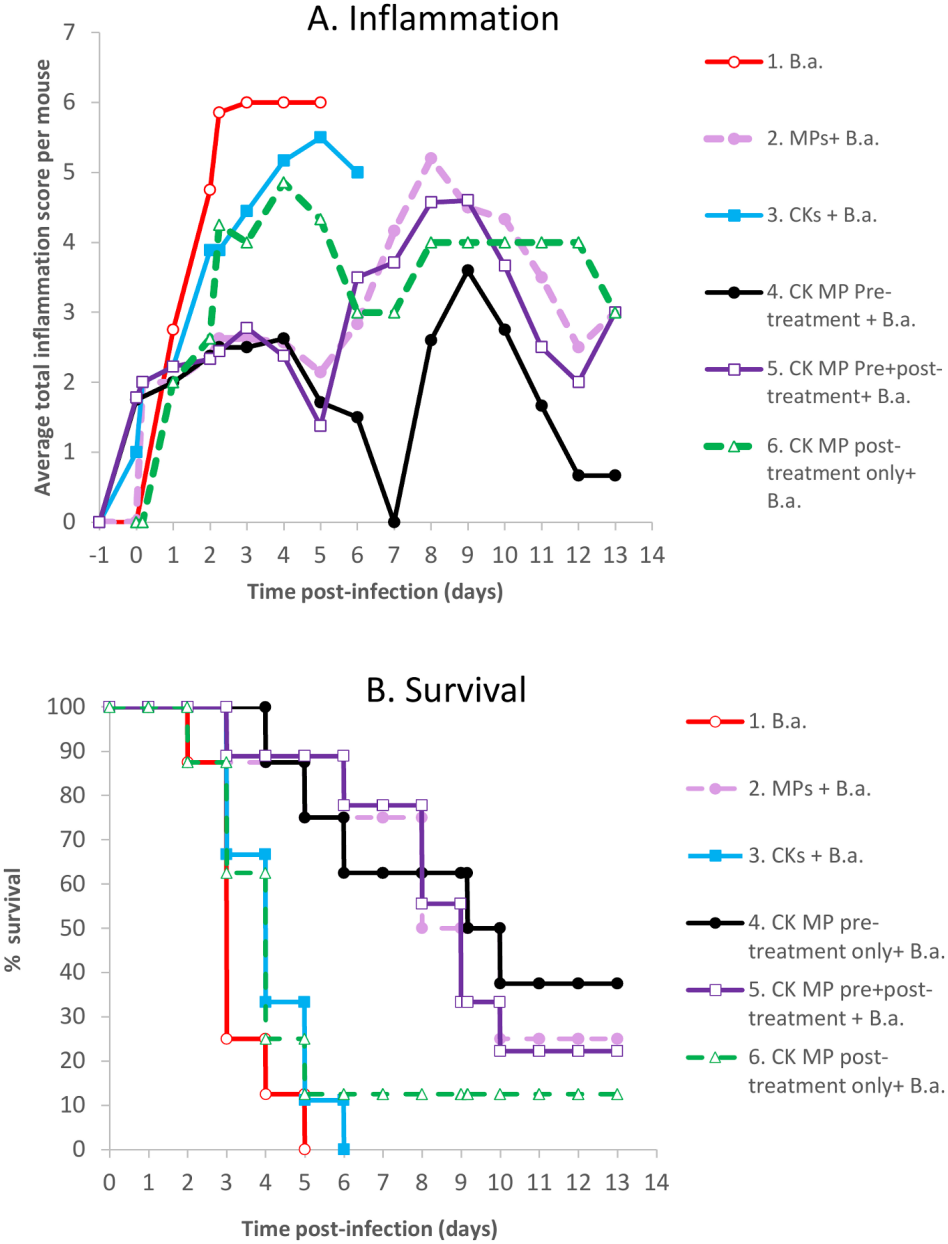
Effect of MP administration on the inflammatory response at the site of *B*.*a*. infection and survival of the spore-challenged mice. Six groups of animals (*n* = 8–9 each) were challenged with equal doses of spores (2.6x10^6^ per 50 μl dose) into each of the hind footpads and treated as following by the same inoculation route. Group 1 (solid line, open circles) served as untreated controls. Group 2 (dashed line, closed circles) received MPs only without CKs at 24 h before (pre-treatment) and two injections at 4 h and 24 h after infection (post-treatment). Group 3 (solid line, closed squares) was pre-and post-treated with a mixture of the soluble CKs (50 ng of each CXCL8 and CCL3 per injection) without MPs. Group 4 (solid line, closed circles) was pre-treated with the same amount of CKs loaded onto MPs, while Group 5 (solid line, open squares) was additionally post-treated with the same MPs. Group 6 (dashed line, open triangles) received an equal dose of CK-loaded MPs as post-treatment injections only. (A) Total scores for inflammation and swelling seen in the challenged footpads per number of mice observed at the indicated time during the course of disease. (B) Kaplan-Meier mortality curves.

The untreated mice in Group 1 developed high level of inflammation and died within 5 days post infection (p.i.) (Fig 2, solid lines with open circles). Similar behavior was displayed by the Group 3 which received soluble CKs. Only marginal reduction of inflammation and delay in mortality were detected (Fig 2, solid lines with closed squares). However, the Groups 2, 4, and 5 corresponding to the administration of MPs and MP loaded with CKs demonstrated substantial differences from the control groups 1 and 3. The average footpad inflammation per mouse in Group 4 showed a biphasic curve with two statistically significant peaks around days 3 and 9 p.i. (p<0.01, α = 0.05) (Fig 2A). The host response was initially reduced, reached minimal levels and then increased again, followed by a final reduction in surviving mice. Groups 2 and 5 appeared visually to progress in a similar biphasic manner, but the difference between the low and high points of the corresponding curves only reached statistical significance in Group 5 during the second phase. Overall, in all groups the death predominantly took place in mice with high inflammatory scores (S1 Fig).

The most prominent effect was observed in the case of CK-loaded MPs administered as a single pre-treatment dose. Statistical analysis using Mann-Whitney U-test confirmed high significance of the inflammatory score differences between Group 1 and Groups 2, 4, 5 at days 2 and 3 (*p* values in the range from 0.001 to 0.008). The MPs without loaded CKs (Group 2, dashed lines with closed circles) also displayed the anti-inflammatory effect during the first phase; however, the level of inflammation was reliably higher than in the case of Group 4 (solid lines with closed circles) during the second phase (*p* = 0.008 at day 7). Fig 2B shows the MP administration in groups 2, 4, and 5 had a strong positive impact on survival in comparison with Group 1 (p<0.0007, Log-Rank test). Group 4 demonstrated the best survival rate of 35% at day 13 and delay in death which correlated with the lowest inflammatory score. However, the differences between survival in Groups 2, 4, and 5 did not reach statistical significance. In contrast to the treatment groups involving the MP pre-treatment, the post-treatment with CK-loaded MPs in Group 6 (dashed line with open triangles) shortly after the spore challenge did not increase survival (Fig 2B). The inflammation during the first phase was high, and all animals (except one) died within 5 days.

The beneficial effects of pre-treatment with CK-loaded MPs on survival and the associated host response were confirmed in the independent experiment replicating Group 1 (*B*.*a*.) and Group 4 (24-h pre-treatment plus *B*.*a*.) which additionally included the 4-h pre-treatment plus *B*.*a*. group. At day 13, 50% and 70% of animals survived in the 4-h and 24-h pretreated groups, respectively. In both groups the inflammatory response displayed a bi-phasic behavior (not shown). The Log-Rank test showed that all three pre-treatment only groups did not statistically differ from each other (*p*<0.21) with the average survival rate of 53±16% (SD).

### The activity of CK-loaded MPs correlates with the reduction of bacterial burden and influx of neutrophils to the sites of infection and regional LNs

**Fig 3:**
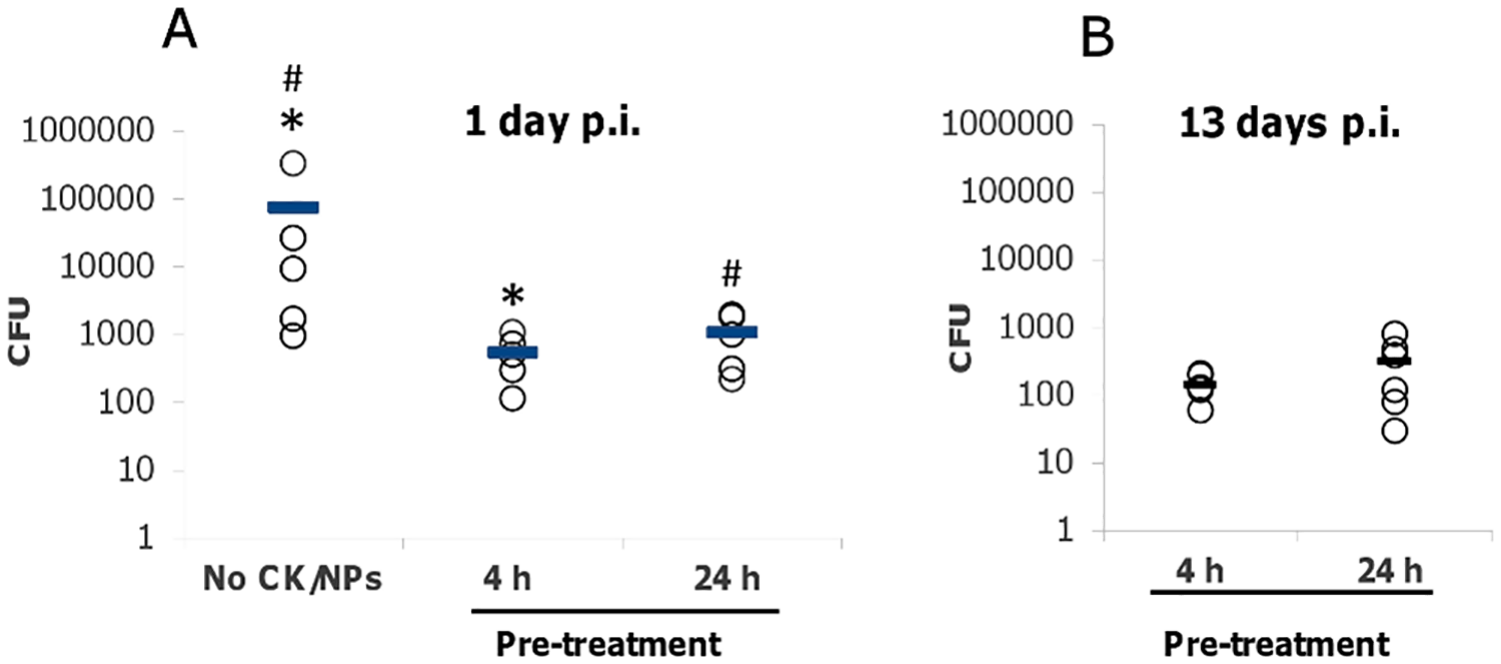
Bacterial load in LNs of mice pre-treated with CK-loaded MPs and control mice at 1 day (A) and 13 days (B) post challenge with 4x10^6^ of *B*.*a*. spores in 20 μl of PBS per hind footpad. Bars indicate arithmetic means in each group. Mann-Whitney U-Test, * *p* = 0.02, # *p* = 0.14, *n* = 4–6 per group.

To determine whether a protective effect of CK-loaded MPs was associated with the reduction of bacterial load, animals were challenged with spores and euthanized at 24 h p.i. The popliteal LNs were surgically removed and plated onto LB agar after homogenization in PBS. Pre-treatment with CK-loaded MPs demonstrated a statistically reliable (*p* = 0.02) decrease in the number of colony-forming units (CFUs) in comparison with the infected controls untreated with MPs (Fig 3A). The duration of pre-treatment (4 h *vs*. 24 h) did not show a substantial difference in the anti-bacterial effect (*p* = 0.14). Surviving mice at day 13 demonstrated low numbers of residual bacteria in LNs, spleen and footpads (Fig 3B and data not shown) indicating that overall the immune response was able to control bacterial propagation and systemic dissemination, but the elimination of persisting bacteria was slow.

**Fig 4:**
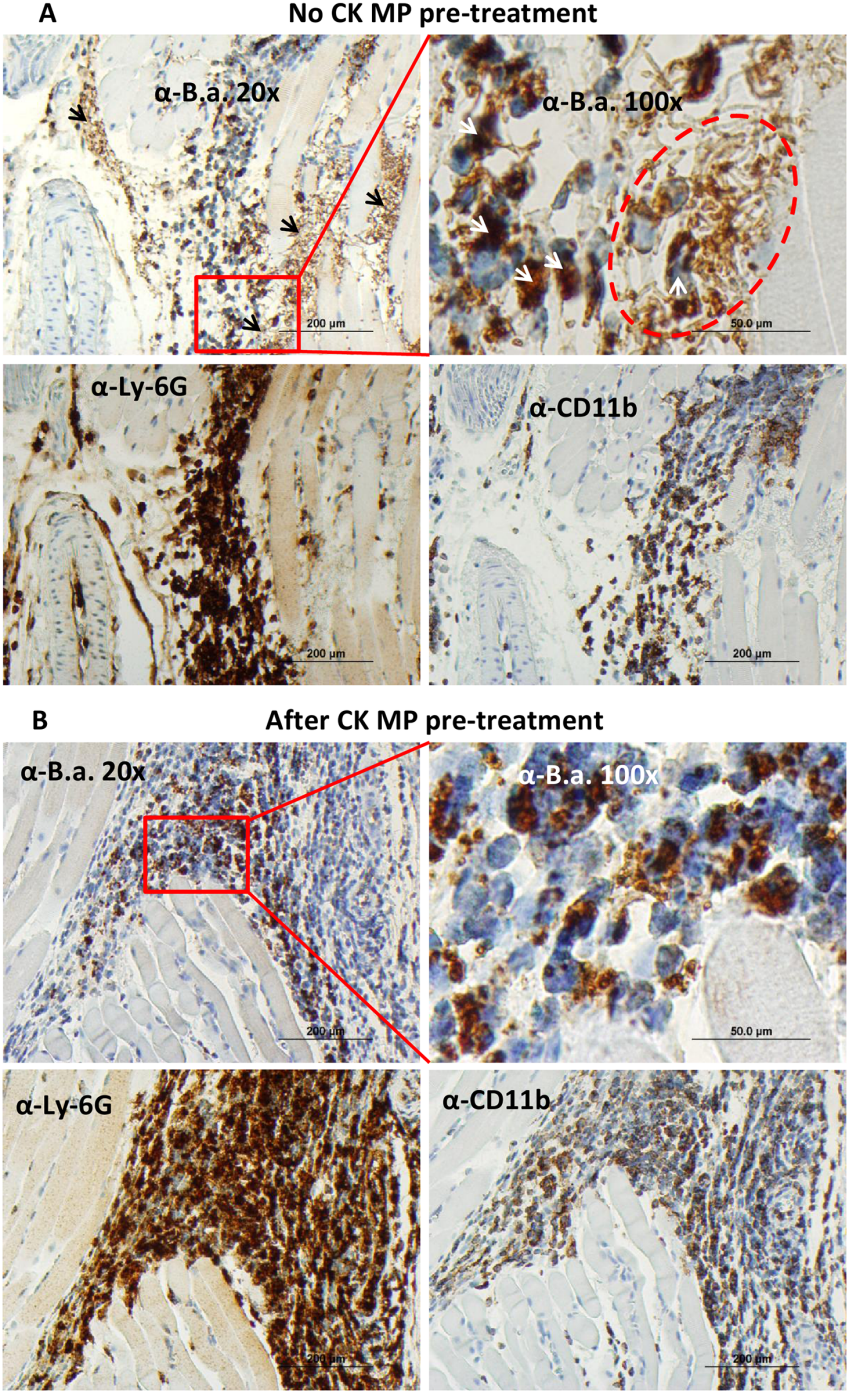
Administration of CK-loaded MPs results in the phagocytic elimination of bacterial burden in the spore-challenged footpads. (A) *B*.*a*. infection with spores (4x10^6^ per hind footpad in 20 μl of PBS) after 24 h shows presence of extracellular bacterial chains and intracellular bacterial antigen (brown stain indicated by black and white arrows, correspondingly; top panels). Red square in the left panel identifies a position of a bacterial swarm shown under magnification in the right panel (dotted line) next to the *B*.*a*.-positive phagocytes (brown color). Consecutive slices of tissue stained with α-Ly-6G and α-CD11b (A, bottom panels) demonstrate infiltration of phagocytes (brown color) to the site of infection. (B) Pretreatment with CK-loaded MPs for 24 h stimulates an elimination of extracellular bacteria. No extracellular bacteria can be found in the infected tissue (top panels). Red square in the left panel identifies a region magnified in the right panel. Multiple phagocytes stained positive for the *B*.*a*. antigens (brown color) are visible. Compared to (A), the consecutive slices of tissue stained with α-Ly-6G and α-CD11b antibodies demonstrate increased infiltration of phagocytes.

Immunostaining of the tissue sections using α-*B*.*a*. serum confirmed the above results. Mice challenged with spores without the MP pre-treatment showed intense staining of the footpad tissue sections which was substantially reduced in mice pre-treated with the CK-loaded MPs (S2 Fig). Further analysis with a neutrophil-specific α-Ly-6G antibody [17] showed that the antibacterial effect of MPs was accompanied by the influx of neutrophils to the site of MP injection (S3 Fig). The *B*.*a*.-positive staining was associated with the extracellular bacterial chains as well as the cytoplasmic content of the infiltrating immune cells (Fig 4A), indicating that these cells were involved in the phagocytic engulfment of bacteria. Staining with the α-Ly-6G and α-CD11b antibodies (specific mainly to neutrophils and monocytes/macrophages, respectively) confirmed a large number of infiltrating phagocytes attracted to the sites of bacterial proliferation (Fig 4A). In contrast to the control infected mice, no extracellular bacteria were visible in the tissue of the CK-pre-treated mice (Fig 4B). The *B*.*a*.-specific intracellular staining overlapped with a large area strongly positive for neutrophils and the less intensely stained macrophages.

**Fig 5:**
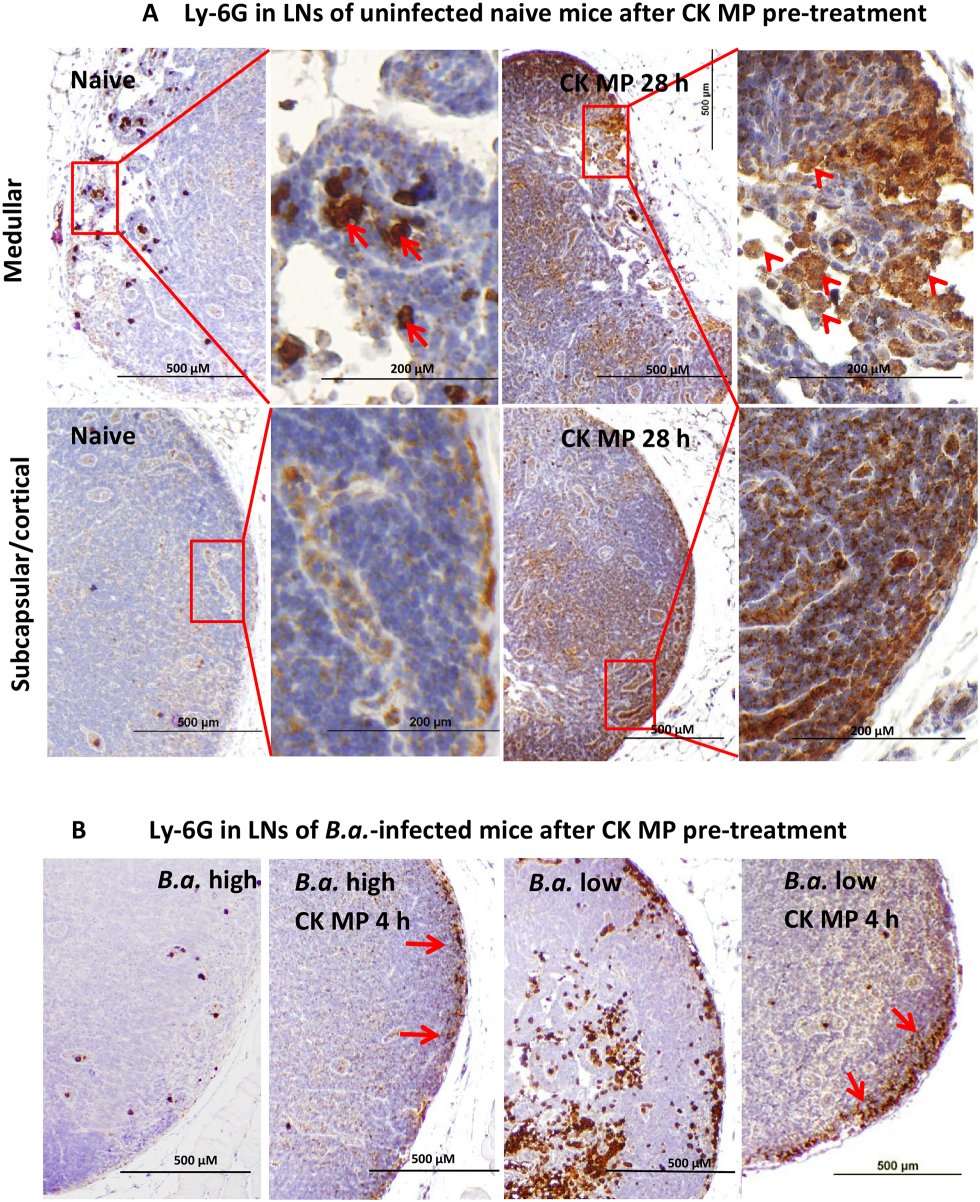
Administration of CK-loaded MPs results in the increased appearance and altered distribution of the neutrophil-specific antigen Ly-6G in the popliteal LNs of naïve and *B*.*a*.-infected mice. (A) Mice were injected into footpads with CK-loaded MPs (CK MPs) for 28 h and the presence of Ly-6G+ cells was revealed immunohistochemically (brown color) using primary antibody against Ly-6G. The medullar (A, top row) and subcapsular/cortical LN regions demonstrate change in the neutrophil antigen distribution pattern in response to CKs. The magnified squared regions in the right panels show the cell stained with high and low intensities (shown by arrows and arrowheads, respectively). The cortical LN regions from the CK-treated mice (A, bottom row) contain a large amount of diffusely-distributed Ly-6G antigen with a pattern shown in the magnified squared region in the right panel. (B) Mice were injected into footpads with CK-loaded MPs (CK MPs) for 4 h and then challenged with *B*.*a*. spores (low, 4x10^5^ spores or high, 4x10^6^ spores in 20 μl of PBS) for 24 h. The neutrophils were stained as in (A). The infection after CK administration resulted in the enhanced number of neutrophils in the subcapsular region (arrows). Low *B*.*a*. dose stimulated the neutrophil migration (B, middle right panel), but high dose abrogated it (B, left panel).

We previously found that a fraction of the spores injected into footpads quickly disseminate to the regional draining LNs where the proliferating bacteria cause extensive pathological changes [15,16]. Immunohistochemical analysis of the LN tissue showed a remarkable change in the neutrophil staining pattern in response to MP-directed CK administration (Fig 5). The high-intensity, punctate staining of occasional neutrophils in naïve mice (Fig 5A, arrows) was replaced after CK administration with a massive diffuse infiltration by the immature neutrophils with lower levels of Ly-6G (Fig 5A, arrowheads). The latter are visible in the high endothelial venules of naïve and CK-pre-treated mice and therefore seem to originate from the blood (Fig 5A, bottom panels). The infectious process stimulated the migration of activated (Ly-6G-high) neutrophils into LNs, but was detected only at low spore dose (Fig 5B, middle right panel), in agreement with the inhibition of neutrophil function by *B*.*a*. [11,18–21]. This immunosuppressive effect was overcome by the CK-pretreatment, which led to the accumulation of neutrophils in the subcapsular space (Fig 5B, arrows in middle left and right panels). Finally, the surviving mice demonstrated the enlarged LNs, the *B*.*a*.-positive staining and the increased presence of neutrophils in the LN germination centers as evidence of the protective immune response (S4 Fig).

## Discussion

This study for the first time demonstrates a protective effect of MPs loaded with CKs against anthrax infection in mice, even in the absence of antibiotic therapy. Our MPs represent a versatile platform consisting of a hydrogel with covalently coupled bait capable of reversible CK binding with a desired affinity and release rate. These parameters can be modified by employing a spectrum of available baits of different nature. In the case of MPs with the triazine dye bait such as CB used in the current and previous studies, the negatively charged sulfate groups of the bait readily bind positively charged CKs. Our data show the binding affinity to be in the range of 0.5 to 5 μM [8], which compares favorably with the CK-extracellular matrix interactions in the natural gradients. The release rate estimates show that a sustained delivery of CKs can be continued for >30 h, sufficient for initiation of LN responses, in contrast to a bolus injection of a biomolecule which is expected to dissipate rapidly, failing to provide the spatial context needed for CK-mediated cell migration. Our MPs are characterized by low toxicity and long circulation rates because a polyacrylamide gel is biologically inert [22–24] while the reactive dyes become virtually non-toxic after chemical coupling to substrates [25]. The process of loading can be carried out in mild physiological conditions eliminating major obstacles of many previous MP designs. As shown in Fig 1 the CCL3 released from the MPs displayed the chemotactic activity corresponding to the fully active CK while the control one in solution decreased its potency upon incubation likely due to aggregation [14]. All chemotactic activity after the loading step was found associated with MPs, making a separation of MPs from the unbound CKs unnecessary. The size of our MPs mimicked microbial cells to ensure co-localization of the MPs and bacteria. Although some data indicate that the drainage of the sub-micron MP to the lymphatics can be restricted [26], we found that a significant portion of the MPs rapidly entered the local LNs *via* afferent lymphatic vessels [27]. In future experiments we are going to optimize the MP delivery and protective effect depending on the particle size.

To study the capacity of the MPs to attract neutrophils, we chose to load the MPs with human CXCL8. This CK plays a dominant role in stimulating neutrophilic inflammation in humans. Although mice do not express CXCL8, they possess a receptor homologous to human CXCR2 that is able to mediate neutrophil chemotaxis in response to human CXCL8 [28–30]. CXCL8 demonstrates high level of neutrophil recruitment in contrast to the endogenous mouse analogs MIP-2 and KC [9]. Anticipating an increased combined effect of CKs belonging to different families, we decided to load the MPs with mouse CCL3 in addition to CXCL8. The CC chemokines were originally described as preferential chemo-attractants and activators of mononuclear cells and eosinophils; however, several studies demonstrate that this chemokine subtype is highly active in stimulating neutrophil migration in murine models [10,31,32].

Antimicrobial neutrophils represent an attractive choice for MP-directed manipulation with their chemotactic behavior. Neutrophils are quickly recruited to the site of infection where they mediate effective bacterial clearance *via* different mechanisms, including the release of lytic enzymes, production of reactive oxygen intermediates, and neutrophil extracellular traps [33–35]. The protective role of neutrophils was demonstrated in several infections [36–48]. However, imbalance in the recruitment and removal of these cells can result in pathologies, including chronic infections and inflammatory disorders [49–51]. Neutrophils from humans and mice are capable of killing *B*. *anthracis* spores and/or bacilli *in vitro* [52–54]. However, the functional chemotactic responses by neutrophils *in vivo* required to exert their bactericidal effect seem to be inhibited by *B*.*a*. [21,55]. Therefore, neutrophils may appear to be unimportant in the defense against *B*.*a*. infection [56]. We hypothesized that timely stimulation of neutrophils as key mediators of innate immunity early in the course of infection would be able to interfere with the suppression of their function and ultimately improve the outcome of disease.

We found that extensive inflammation of footpads in the spore-challenged control group was followed by rapid onset of mortality (Fig 2). However, in the groups of mice which received the MP-based treatments before spore challenge the inflammation was reduced while the mortality was delayed and/or partially prevented. The most prominent anti-inflammatory effect, which took place during the first and second phases of inflammation, was observed in Group 4 treated with CK-loaded MPs. In comparison, the MPs without CKs in Group 2 also demonstrated similar reduced response during the first phase of inflammation, which subsided at day 7 in Group 4 but remained significantly higher in Group 2 (*p* = 0.008). A different result was obtained in the case of the Group 6 treated with CK-loaded MPs shortly after the spore challenge. This group showed high mortality and inflammation close to that of untreated control which is consistent with quick systemic dissemination of the spores through lymphatics to distant locations such as the spleen within less than 3 h [27]. This would render the LN-attracted neutrophils ineffective in eliminating the remote spores.

Several explanations can be put forward for the observed protective effects. We show that both the administration of CK-loaded MPs and spore challenge resulted in the migration of neutrophils to the site of inoculation and regional popliteal LNs as documented immunohistochemically by specific neutrophil marker Ly-6G. Additionally, many migrating monocytes/macrophages were also present, as shown by the CD11b-positive staining (Fig 4). We suggest that prophylactic administration of CK-loaded MPs before the spore challenge effectively reduced the bacterial burden due to the activity of phagocytes demonstrating large amount of *B*.*a*. antigen in their content (Fig 4B). The short 4-h pre-treatment was statistically as effective as the 24-h one, indicating quick and sustained mobilization of circulating neutrophils. Judging by the predominant number of Ly-6G+ cells compared to CD11b+ ones, the majority of cells recruited were resting neutrophils (CD11b^-^Ly6G+), which can be activated by CXCL8 to become CD11b+ [51,57].

During the first phase the MP-delivered CKs seem to overcome the suppressive effect of *B*.*a*. infection on the actin-based neutrophil chemotaxis. Even a low level of neutrophil influx in response to MPs without CKs reported in our previous study [8] seems enough to shift the course of *B*.*a*. infection toward clearance by host immune cells. Our preliminary data (not shown) indicate that the MPs do not display a direct anti-spore or anti-bacterial activity but can be involved in the induction of endogenous CKs such as Gro/KC which can work in concert with the externally-delivered CKs during the first phase of the host inflammatory response. This suggestion is consistent with observation of low-level neutrophil migration to the site of subcutaneous injection of agarose beads coupled with CB [58].

The appearance of a second peak of inflammation might be at least partially attributed to the waning of the CKs’ effect as their sustained release according to our estimates can take place only for a few days. It is likely that the decreased innate response during the second phase was not replaced by the fully protective adaptive immunity and therefore could not prevent death. Some of the surviving mice still demonstrated footpad inflammation and residual bacterial burden. Experiments in this direction are forthcoming.

Overall, we demonstrated a remarkable effectiveness of CK-loaded MPs which resulted in a substantial delay in mortality and improvement in survival after a single injection of MPs without any additional therapeutic intervention. Although potential clinical utility of our MPs requires further studies, the presented data suggest that stimulation of the immune responses with exogenous CKs may be considered as novel strategy to improve outcome of anthrax infection. In future experiments it seems promising to combine our MP-based delivery with administration of antibiotics for increased efficacy, or to apply this approach to other types of diseases.

## Supporting Information

S1 Fig Mice that succumbed to infection demonstrate elevated levels of footpad inflammation. (DOCX)

S2 Fig Administration of CK-loaded MPs reduces bacterial burden in the spore-challenged footpads. (DOCX)

S3 Fig Administration of CK-loaded MPs and spore challenge induce migration of neutrophils to the inoculation site. (DOCX)

S4 Fig LNs of surviving mice demonstrate size enlargement and the appearance of follicular zones which stain positive for Ly-6G and *B*.*a*. antigens. (DOCX)
