# Supplementary material for: Chemokine-Releasing Microparticles Improve Bacterial Clearance and Survival of Anthrax Spore-Challenged Mice
Source: PLoS One. 2016 Sep 15;11(9):e0163163. doi: 10.1371/journal.pone.0163163 (PMC5025034; doi:10.1371/journal.pone.0163163)
Supplement: S1 Fig — (DOCX) [file pone.0163163.s001.docx]

**Fig. S1. Mice that succumbed to infection demonstrate elevated levels of footpad inflammation.** Each data point indicates the total inflammation score for one or several mice with the same score at the particular observation time. Higher inflammation scores were not a direct predictor of mortality as some mice demonstrated greater inflammation and survived or died at a later time, but all mice which died in any group demonstrated increased inflammation.
