# Supplementary material for: Chemokine-Releasing Microparticles Improve Bacterial Clearance and Survival of Anthrax Spore-Challenged Mice
Source: PLoS One. 2016 Sep 15;11(9):e0163163. doi: 10.1371/journal.pone.0163163 (PMC5025034; doi:10.1371/journal.pone.0163163)
Supplement: S2 Fig — (DOCX) [file pone.0163163.s002.docx]

**Fig.S2. Administration of CK-loaded MPs (CK MPs) results in the reduction of bacterial burden in the spore-challenged footpads.** Mice were challenged with *B.a.* spores (4x10^6^ per hind footpad) without pre-treatment (C-D) or after pre-treatment (E-H) with CK MPs for 4 h (E, F) and 24 h (G, H). At 24 h p.i. the mice were euthanized and footpads prepared for immunohistochemistry with α-*B.a.* serum. Stained bacteria are colored brown. Two representative fields of view are shown for each condition.
