# Supplementary material for: Chemokine-Releasing Microparticles Improve Bacterial Clearance and Survival of Anthrax Spore-Challenged Mice
Source: PLoS One. 2016 Sep 15;11(9):e0163163. doi: 10.1371/journal.pone.0163163 (PMC5025034; doi:10.1371/journal.pone.0163163)
Supplement: S3 Fig — (DOCX) [file pone.0163163.s003.docx]

**Fig.S3. Administration of CK-loaded MPs (CK MPs) and spore challenge induce migration of neutrophils to the inoculation site.** Naïve mice (A) were injected into hind footpads with CK-loaded MPs for 4 h (B) and 24 h (C) and then challenged with *B.a.* spores (4x10^5^ per hind footpad) (D to F, correspondingly). At 24 h p.i., mice were euthanized, soft tissue from the footpads was removed for the preparation of slides. The presence of neutrophil marker Ly-6G was revealed immunohistochemically (as a brown color of diaminobenzidine stain) using primary antibodies against Ly-6G.
