# Supplementary material for: Chemokine-Releasing Microparticles Improve Bacterial Clearance and Survival of Anthrax Spore-Challenged Mice
Source: PLoS One. 2016 Sep 15;11(9):e0163163. doi: 10.1371/journal.pone.0163163 (PMC5025034; doi:10.1371/journal.pone.0163163)
Supplement: S4 Fig — (DOCX) [file pone.0163163.s004.docx]

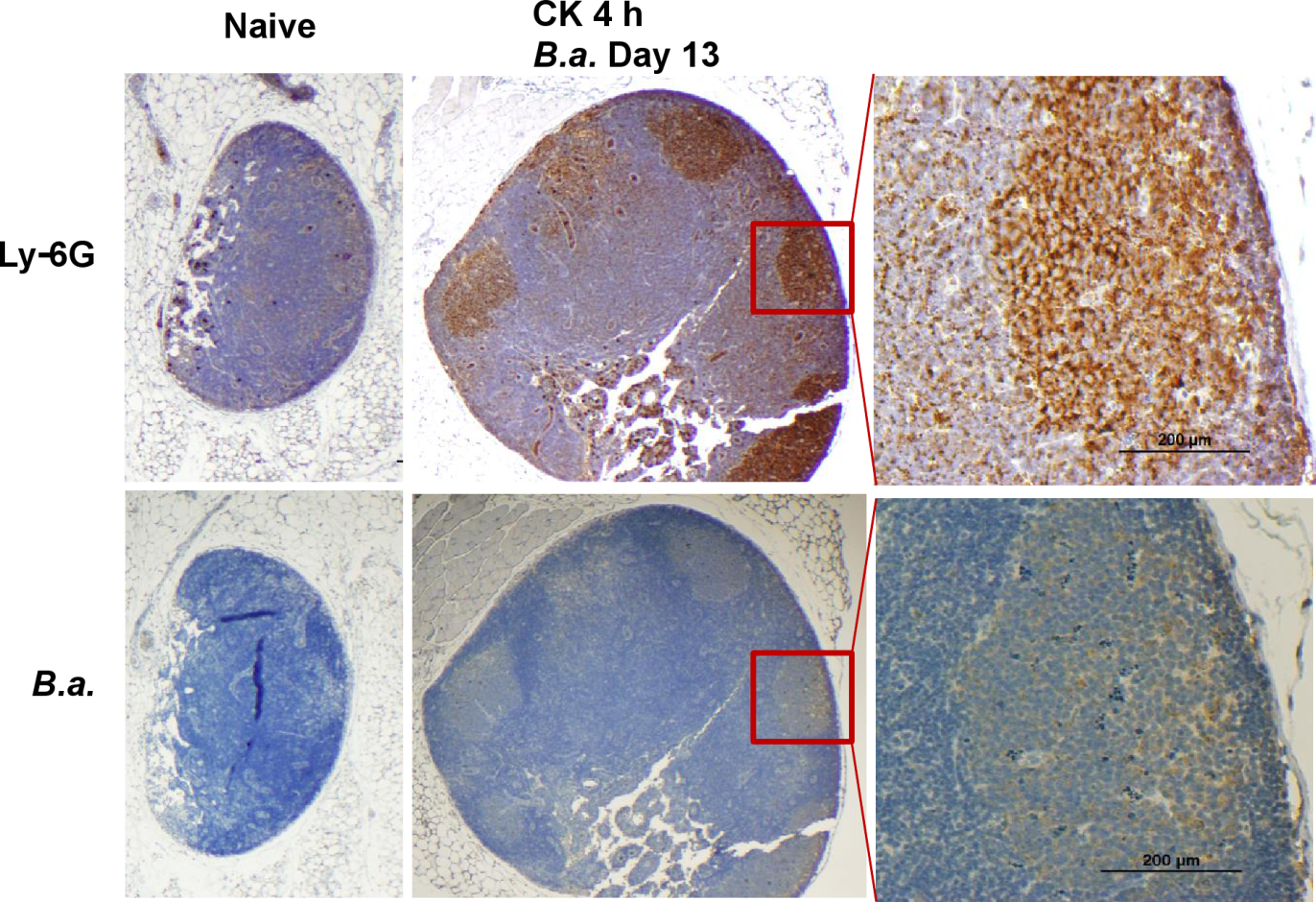


**CK MPs**

***B.a.* Day 13**

**Fig.S4. LNs of surviving mice demonstrate size enlargement and the appearance of follicular zones which stain positive for Ly-6G and *B.a.* antigens.** Mice were injected into hind footpads with CK-loaded MPs (CK MPs) for 4 h and challenged with *B.a.* spores (2.6x10^6^ per hind footpad). Naïve mice were left untreated and unchallenged. After 13 days, mice were euthanized, LNs removed and used for the preparation of slides. The presence of neutrophil marker Ly-6G and *B.a.* antigens were revealed immunohistochemically (as brown color of diaminobenzidine stain) using the consecutive slices of tissue and primary antibodies against Ly-6G and immune serum against *B.a.*, correspondingly. Right panels magnify areas marked in the middle panels with red boxes.
